# The impact of integrated quality management-based health services on general hospital quality

**DOI:** 10.3389/fpubh.2022.1011396

**Published:** 2022-09-20

**Authors:** Nur Hidayah, Arbianingsih, Ilham

**Affiliations:** ^1^Nursing Management Department, Faculty of Medical and Health Sciences, State Islamic University of Alauddin, Makassar, Indonesia; ^2^Faculty of Medical and Health Sciences, State Islamic University of Alauddin, Makassar, Indonesia; ^3^Faculty of Islamic Economics and Business, State Islamic University of Alauddin, Makassar, Indonesia

**Keywords:** total quality management, hospital, health services, patient care services, public health

## Introduction

The global trend toward Total Quality Management (TQM) implementation aims to steadily improve the quality of healthcare services to fulfill patients' expectations and maximize the use of available resources to improve care outcomes. Utilizing TQM processes in the healthcare sector can increase patient safety. TQM has emerged as a promising method for boosting the effectiveness and efficiency of health care demand in this respect (1). TQM emphasizes people and processes. Its goals are organizational success and customer satisfaction (2). Increases in health care expenditures, reliance on technology, and the need to meet international standards and licenses are some of the primary difficulties facing current health organizations (3), and meeting the demands of patients, which necessitates hospitals to maintain a high standard of service.

TQM improves hospital performance (4–6). Several studies have been conducted to investigate the effects that putting TQM principles into practice has on an organization's overall effectiveness and performance. A great number of studies have uncovered significant and favorable associations (7). In boosting hospital quality, the phenomenon of inadequate implementation of comprehensive quality management is a major worry for hospital executives and personnel in general hospitals. Despite having a hospital disaster plan and conducting drills and simulations, it has been discovered that public hospitals are unprepared and vulnerable to man-made catastrophes. All of these factors can affect the hospital's capacity to provide patients with quality and safe care (8).

This research's objective was to examine the role of integrated quality management of health services in enhancing hospital quality. When doing research, the researchers examined the literature as a methodology. In this research, the content analysis method was utilized as the approach to data analysis that was utilized (9). This research may aid healthcare firms in implementing a more efficient TQM approach. It will encourage general hospitals to provide high-quality patient care services.

## Integrated quality management

TQM includes everyone in the enhancement of processes, goods, services, and culture in order to satisfy consumers and other stakeholders (10). TQM emphasizes customer demands and expectations to enhance product/service/process quality and business performance (2). Increasingly, TQM is becoming recognized as a critical component of more ethical business practices and the long-term growth of human capital. TQM-based governance, especially in terms of improving the quality of the company. Participation is mandatory for all members of the organization. The advancement of business management, production management, marketing management, customer service and management, human resources, and financial resources are all dependent on it. In a TQM system, organizational leaders understand that the organization is a system, help employees grow, set up multiple ways for different levels of the organization to talk to each other, and use the information to make good decisions. Leaders should also encourage employees to take part in making decisions and give employees some control over their jobs. The level of dedication and participation shown by upper management is among the most important factors that can be considered when analyzing the efficiency of TQM procedures. To increase employees' understanding of quality activities in TQM adoption and practices, managers should exhibit more leadership than conventional management behaviors (2, 11, 12).

TQM's guiding principles are continuous improvement, management commitment to customer happiness, employee empowerment, and customer focus (13). Numerous fresh improvement projects are founded on TQM concepts, despite the belief that TQM is an antiquated notion. For instance, the well-known Six Sigma concept for achieving zero errors is not an alternative to overall quality management but rather a methodology that is included in it. The TQM is to make it possible for an organization to offer goods and services of the highest possible quality. This will allow the organization to be more competitive and perform better (14). In addition to this, research has shown that an increase in quality has a beneficial effect on the overall performance of an organization (15). TQM has the potential to decrease errors and improve patient satisfaction. Specifically, TQM will facilitate the development of a patient-centered, safe and effective system, thereby enhancing patient satisfaction (6, 16, 17).

With TQM, the goal is to engage and motivate all levels of the organization's workforce to take ownership of the company's success. It is essential that everyone in the organization work together to continuously improve the targeted solution, namely, the quality of care provided to patients in order to meet their needs and expectations (17). TQM is essential for an organization to enhance service quality and resource utilization (1). In international competition, organizations improve their global competitiveness by providing high-quality products and services (18). The first nation to implement the procedure was Japan. However, the application's theoretical concepts were developed in the United States. During the middle of the 1980's, TQM gained popularity. On the other hand, the majority of the ideas that form the basis of the TQM principles were developed in the 1950 and 1970's (6, 19).

## Hospital quality

The continual advancement of science and technology, along with an unrelenting focus on improving patient care, are the defining characteristics of the processes used to evaluate hospital quality (20). Quality has become increasingly important to businesses like hospitals and other sectors with significant customer bases (6, 21). The patient's expectations before they make a decision can have an impact on the quality of the service they receive, as can the quality that is provided and the quality of the output that is received. When evaluating the quality of care provided to a patient, one must start with the patient's requirements and proceed to evaluate the patient's level of satisfaction. Both the patients' expectations and their actual experiences shape the quality of the service they receive. If the perceived service matches the expected service, the quality will be excellent or positive. When compared to what was anticipated, the actual level of service provided is judged to be superior to the ideal. If the actual quality of the perceived service is lower than what was anticipated, then the overall quality of the perceived service is considered to be negative or poor (22).

Positivity among customers is directly related to the level of service they receive, which in turn is determined by their subjective opinions about how well they feel they were treated and how well they feel they received their desired results (23). High-quality care is defined as meeting the needs of patients while also satisfying the requirements of healthcare providers by adhering to the standards and guidelines that have previously been established in the clinical setting (24). Customers are more likely to remain loyal to a company if they experience high levels of satisfaction with the brand and the products or services they receive (25).

A concerted effort on the part of health care staff and stakeholders to address system-wide problems is required in order for there to be improvements in the overall quality of hospitals. The first thing that needs to be done is to put together a maintenance crew that is capable of providing high-level problem care, effective leadership, and adaptable change management (26). Several aspects go into determining the quality of the service provided, and they are as follows: (i) Delivering on promises, providing accurate service from start to finish, and handling issues with dependability are all examples of excellent customer service; (ii) Service that is delivered quickly and appropriately, ready response, and all-around consumer help measure responsiveness; (iii) Trust, safety, and friendliness are all guaranteed; (iv) Physical form is evaluated using Phys. The result of this is that hospital-based quality indicators, which are frequently linked to the hospital's structure, process, or outcomes, always evaluate the quantitative and/or qualitative care provided. The indicators provide a description of particular facets of healthcare that are utilized for the purposes of monitoring, benchmarking, and prioritizing activities in order to accomplish continuous quality improvement (27, 28).

## Discussion and opinion

When determining the quality of a product or service, TQM takes into account both internal and external customer feedback. Therefore, for hospital-affiliated parties to comprehend and value what it means for quality to be present, they must first gain an understanding of both the process and the customer. In TQM, all of a company's management activities are geared toward achieving one primary goal: customer satisfaction. Regardless of the actions taken by management, they will be ineffective if they do not ultimately increase the level of customer satisfaction. When it comes to the increasingly cutthroat competition that exists between the managers of health care services, the pursuit of quality is at the forefront of the conversation. As a consequence of this, the TQM methodology places a high priority on accurately determining the requirements of customers as a component of the process of coming up with a new product or service.

With the help of TQM, managers can offer strategic solutions that focus on prevention rather than inspection; thus, it can also be used as a detailed strategy to develop organizational effectiveness that involves everyone involved in the process (6). It is necessary to examine the policymakers or stakeholders who were involved in the structural and functional preparation of the vision and mission structure and function in order to support the evaluation of the implementation of health services based on the existing vision and mission. This will help ensure that the evaluation is accurate. One of the indicators of a good management system is the degree to which the implementation is based on the vision and mission. In most cases, hospitals will adhere to a quality policy, which is also sometimes referred to as a service commitment to patients, in order to work toward the objective of achieving high levels of patient satisfaction. It is anticipated that quality policies in hospitals will include not only the systems that support services to customers/patients but also the systems that implement health and work safety in hospitals and other social systems. This will be the case because quality policies in hospitals will include the systems that support services to customers/patients.

A comprehensive system that identifies and verifies all aspects and elements that facilitate implementation in accordance with standards is required to support the quality of a hospital's care as it is provided to patients. This is necessary in order to maintain the high level of care that the hospital provides. Hospitals are required to plan out a work program in a manner that is reflective of the quality goals that the hospital has set for itself. TQM is also seen as an effective and well-integrated way to develop, improve, and keep quality high. This enables all departments to perform at the highest possible level for the lowest possible cost in order to fulfill the requirements of customers (29, 30). TQM practices are needed for successful implementation and improved performance, according to studies (30, 31). TQM is a management approach that is widely regarded as being forward-thinking and innovative among both businesses and other types of organizations. TQM is a system that, when applied to the medical industry, ensures that a quality focus is maintained throughout every step of the healthcare delivery process. This ensures that patients receive the highest possible standard of care at all (32).

There is a correlation between patient satisfaction ratings and technical measures of care, which indicates that these metrics can be used to evaluate the quality of care provided by a hospital as a whole (33). If it is implemented, TQM will lead to increased levels of nurse performance at every level (32, 34). In addition, both theoretical research and data collected from the real world have demonstrated that implementing TQM in an organizational process invariably leads to improvements in that organization's level of performance. This has been shown to be the case in a way that is statistically significant. This is the case regardless of whether the research is carried out in a controlled environment or in the actual environment. The TQM methodology places an emphasis on patient satisfaction, the identification of organization-wide problems, the development and promotion of open decision-making among staff members, and the development and promotion of open decision-making among patients. In addition, the methodology places an emphasis on the identification of organization-wide problems. Each employee is responsible for the quality of the work they produce, and it is done so in a way that makes this possible. This approach allows each employee to take on some of the accountability for the total amount of work completed.

## Conclusion

This study comes to the conclusion that the TQM can be applied to hospital organizations, and that if it is correctly implemented, it has the potential to contribute to an improvement in the quality of hospital care. In addition to that, it ought to provide direction for the implementation of TQM, which addresses errors, boosts quality, and increases patient satisfaction as a result of contrasting the current performance with that of the previous year. This is done in order to ensure that patients receive the best care possible. Because it helps improve the performance of healthcare professionals, TQM is beneficial to the healthcare industry because it leads to higher standards of conduct and more complete dedication to the care of patients. This will, over the course of time, result in an improvement in the overall quality of general hospitals because the quality of hospital programs is dependent on the development of departmental practices toward the establishment of standards. TQM is beneficial to health services because it helps improve the performance of health workers, which results in higher quality behavior and a total commitment to working with patients. TQM is also beneficial because it helps improve the performance of patients. A strategy based on total quality management is utilized in order to achieve this goal successfully. As a result of this, the focus that is placed on subsystems within hospital quality control serves to both introduce and put into practice TQM. One method that can be used to accomplish this objective is the creation of an exhaustive TQM taxonomy. This taxonomy would describe the manner in which TQM practices are integrated into systems that facilitate higher levels of performance, as well as the reasons for doing so.

## Author contributions

NH collected and analyzed the literature. A and I verified the literature findings. All authors jointly completed the discussion of this research.

## Conflict of interest

The authors declare that the research was conducted in the absence of any commercial or financial relationships that could be construed as a potential conflict of interest.

## Publisher's note

All claims expressed in this article are solely those of the authors and do not necessarily represent those of their affiliated organizations, or those of the publisher, the editors and the reviewers. Any product that may be evaluated in this article, or claim that may be made by its manufacturer, is not guaranteed or endorsed by the publisher.
